# Venetoclax in Acute Myeloid Leukemia: Molecular Basis, Evidences for Preclinical and Clinical Efficacy and Strategies to Target Resistance

**DOI:** 10.3390/cancers13225608

**Published:** 2021-11-09

**Authors:** Sylvain Garciaz, Colombe Saillard, Yosr Hicheri, Marie-Anne Hospital, Norbert Vey

**Affiliations:** 1 Aix-Marseille University, INSERM, CNRS, Institut Paoli-Calmettes, 13009 Marseille, France; veyn@ipc.unicancer.fr; 2 Deparment of Hematology, Institut Paoli-Calmettes, 13009 Marseille, France; saillardc@ipc.unicancer.fr (C.S.); hicheriy@ipc.unicancer.fr (Y.H.); hospitalm@ipc.unicancer.fr (M.-A.H.)

**Keywords:** venetoclax, BH3-mimetics, acute myeloid leukemia, apoptosis, mitochondria

## Abstract

**Simple Summary:**

Venetoclax is a BH3-mimetics that specifically inhibits the antiapoptotic protein BCL-2. The drug has considerably expanded the treatment options of acute myeloid leukemia (AML) patients unfit for intensive treatment regimens. In recent phase III trials, combination of venetoclax with the hypomethylating agents azacitidine or with low dose cytarabine, was associated with a 48–65% response rate and a 7.2–14.7 months overall survival. Recent studies also suggest that the drug is highly efficient in combination with high-dose chemotherapy and targeted therapeutics, including IDH and FLT3 inhibitors. In this review, we summarize the molecular basis of BCL-2 inhibition in AML, the effect of venetoclax in preclinical models and clinical studies and discuss the main molecular pathways and therapeutic options for overcoming venetoclax resistance. Identifying new therapeutic strategies increasing BH3 mimetics activity and/or sensitizing toward apoptotic cell death will represent a major challenge for treating AML patients in the near future.

**Abstract:**

Venetoclax is a BH3-mimetics agent specifically interacting with the antiapoptotic protein BCL-2, facilitating cytochrome c release from mitochondria, subsequent caspases activation, and cell death. Utilization of venetoclax has profoundly changed the landscape of treatment for the poor-prognosis category of AML patients unfit for intensive chemotherapy. In the phase III VIALE-A study, Venetoclax, in combination with the hypomethylating agent azacitidine, showed a 65% overall response rate and 14.7-month overall survival, in comparison with 22% and 8 months in the control arm. These results led to the widespread use of venetoclax in this indication. Other combination regimens, consisting of low-intensity, intensive, or targeted therapies are currently under evaluation. Despite promising results, preventing relapses or resistance to venetoclax is still an unmet clinical need. Numerous studies have been conducted to identify and overcome venetoclax resistance in preclinical models or in clinical trials, including the inhibition of other antiapoptotic proteins, the induction of proapoptotic BH3-only proteins, and/or the targeting of the mitochondrial metabolism and machinery.

## 1. Introduction

Acute myeloid leukemia (AML) is a heterogeneous group of diseases with various molecular alterations and mainly occurring after the sixth decade. Treatment modalities of newly diagnosed AML (ND AML) depend on age, fitness, and molecular risk groups based on cytogenetics and the presence of molecular alterations, such as fms like tyrosine kinase 3 (FLT3), nucleophosmin 1 (NPM1), or tumor protein p53 (TP53) mutations, as defined in the ELN 2017 classification [1]. More than 60% of younger patients will be cured by an intensive cytotoxic therapy (ICT) induction based on the association of anthracyclin and cytarabine (CYTA) (7 + 3), followed by consolidations with high doses of CYTA and/or allogeneic stem cell transplantation (HSCT) [2]. However, half of patients >65 years will not be able to receive ICT because of age, poor general status, or comorbidities (unfit patients) [3]. Substantial progress has been made over the past decade, with new therapeutic approaches, including low-dose cytarabine (LDAC) and the hypomethylating agents (HMA) decitabine (DEC) and azacitidine (AZA) [4,5,6]. Although their use has led to significant improvements in the outcome of older, unfit AML patients, results remain unsatisfactory. New therapeutic options, including small inhibitors of somatic mutations commonly found in AML, such as FLT3 or isocitrate dehydrogenase (IDH) inhibitors, have been approved recently for relapsing or refractory (RR) patients. Despite promising results in molecular subgroups, the vast majority of patients cannot benefit from targeted therapies, because of a lack of targetable genetic alterations. In these situations, new combinations or treatments are urgently needed.

## 2. Molecular Basis for BCL-2 Homology 3 (BH3)-Mimetics Drugs in Cancer Therapy

### 2.1. Apoptotic Pathway

Resistance to cell death and dysregulated apoptosis is a hallmark of cancer, discovered more than 50 years ago [7,8]. Mitochondria are the central organelles orchestrating apoptosis, from various stimuli coming from other cells through cell surface receptor binding (extrinsic pathway) or from the cell itself sensing cell stress (intrinsic pathway). One of the major regulators of the intrinsic pathway is the B-cell lymphoma 2 (BCL-2) protein, which was first identified through chromosomal mapping in follicular lymphoma, where constitutive BCL-2 gene expression is driven from the immunoglobulin locus by the t(14;18) translocation [9,10]. Contrary to other known oncoproteins facilitating cell growth, BCL-2 facilitates oncogenesis through resistance to cell death [11,12]. In the following years, more than 15 proteins were integrated into the BCL-2 family, sharing BCL-2–like homology domains 1–4 (BH1-BH4), and with each one playing a specific role in the intrinsic apoptotic pathway. The BCL-2 family can thus be divided into three main groups: antiapoptotic (BCL-2, BCL-XL, BCL-W, BCL2A1, and MCL-1), proapoptotic (BAX, BAK, and BOK), and the BH3-only proteins (BIM, BAD, BIK, BID, BMF, HRK, NOXA, and PUMA), which have also proapoptotic roles. Once activated, BAX and BAK oligomerize, creating pores in the mitochondrial outer membrane, the so-called mitochondrial outer membrane permeabilization (MOMP), with subsequent release of cytochrome C and activation of caspases, and ultimately leading to proteolytic cell death [13]. BCL-2 counteracts this cascade by sequestering proapoptotic proteins and preventing MOMP.

### 2.2. The BH3-Mimetics Class in Cancer Therapy

Given its crucial role in escaping apoptosis in cancer, efforts have been made to develop BH3-mimetic agents and in particular BCL-2 inhibitors. Preclinical data of genetic inhibition of *BCL-2* gene showed promising results in a murine leukemia mouse model [14]. Nevertheless, the first attempts to target *BCL-2* with the antisense nucleotides G3139 in leukemia models were not fully successful, due to the long half-life of the BCL-2 protein [15]. Using an innovative fragment-based approach for drug discovery, combining a chemical screen with the identification of ligands by nuclear magnetic resonance (NMR), followed by parallel synthesis and a structure based design, the first BH3-mimetic, ABT-737, was synthesized in 2005 [16]. ABT-737 binds BCL-2 and BCL-XL with a high affinity, and the drug has shown encouraging preclinical results with in vitro and in vivo models of cancers [16,17,18].

Later, the orally bioavailable ABT-263 (navitoclax), also inhibiting BCL-2 and BCL-XL, was developed [19]. Despite promising results in phase I studies, the dose limiting toxicity of the treatment was thrombocytopenia, as BCL-XL is essential for mature platelet survival [20,21,22]. In an effort to develop a more specific inhibitor, Abbvie and Genentech (members of the Roche group) synthesized ABT-199 (venetoclax, abbreviated to VEN hereafter), a potent, selective, and orally available inhibitor of BCL-2 [23]. VEN binds to the BH3 domain of BCL-2 proteins with the subsequent release of proapoptotic proteins, activation of BAX/BAK, cytochrome c release, and apoptosis induction. Lymphoid malignancies provided the most obvious substrate for this therapeutic, given the frequent overexpression of BCL-2 and the t(14;18) chromosomal rearrangement. The drug induced a strong apoptosis in vitro and a decrease in tumor volume, while sparing platelets in in vivo models of cancers, compared to navitoclax. Of note, a single dose of VEN (200 mg or 100 mg) resulted in clinical response within 24 h and an almost complete reduction in the peripheral blood lymphocytosis associated with tumor lysis syndrome (TLS) in patients with chronic lymphocytic leukemia (CLL) [23]. Rapidly, the FDA and European Medicine Agency (EMA) approved VEN for the treatment of relapsed CLL with 17p deletion [24].

### 2.3. Rationale for Targeting BCL-2 in AML

In AML, unlike in lymphoid malignancies, BCL-2 gene is not rearranged, neither is it always overexpressed, but, surprisingly, this does not precluded BH3-mimetic’s efficacy, illustrating the concept of non-oncogene addiction, by which a cancer cell becomes dependent on a normal cell function [25,26]. BH3-profiling is an in vitro experimental assay able to reveal the dependency on a given antiapoptotic proteins [27]. Indeed, cancer cell survival depends on the sequestration and, therefore, the inhibition of selective proapoptotic BH3-only proteins by antiapoptotic proteins. For example, BIM proapoptotic peptide binds BCL-2, BCL-XL, and MCL-1 antiapoptotic proteins; BAD is linked with BCL-2 and BCL-XL, but not with MCL-1, while HRK and MS-1 bind BCL-XL and MCL-1, respectively [28]. In practice, the cells are treated in vitro with a panel of peptides derived from BH3-only proteins that selectively antagonize the individual BCL-2 family members. The assay measures the amount of cytochrome c released into the cytosol caused by standardized doses of the BH3 peptides. For instance, a mitochondrial response to the MS-1 peptide is an indicator of MCL-1 dependency, whereas mitochondrial response to BAD peptide indicates a BCL-2 dependency. By performing this assay on AML cell lines and primary blasts, A. Letai’s Lab at the Dana-Farber Cancer Institute, Boston, USA, showed that AML blasts were primed for BCL-2, (i.e., BCL2 inhibition lowered the threshold to apoptotic induction), whereas normal HSC were less sensitive to BCL-2 inhibition [27,29,30]. These results were consistent with the lethality of disrupting in vivo the *BCL-2* gene in leukemia mice models and prompted the development of BCL-2 inhibitors in AML [14,31].

### 2.4. BCL-2 Inhibitors in AML, Preclinical Studies

The first generation BCL-2 inhibitor ABT-737 induced apoptosis in AML-derived cell lines, in primary blasts, and in phenotypically defined AML stem cells; inhibited the growth of clonogenic AML progenitor cells; and was effective in reducing the leukemia burden in vivo in mice models of AML [32,33]. The second generation BCL-2 inhibitor VEN induced rapid apoptosis in a variety of AML cell line models at nanomolar concentrations and inhibited leukemia progression in vivo in a murine AML xenograft model, by inducing a short (one week) but significant gain in survival when orally administered at a 100 mg/kg dose [34].

Preclinical works suggested a synergistic activity between BCL-2 inhibitors and AZA. The use of VEN or ABT 737 sensitized both AML cell lines and patient-derived AML samples to the concomitant use of AZA [18,35]. Nevertheless, the rationale for associating the two drugs is not completely understood. AZA does not affect the expression of antiapoptotic proteins but induces the expression of the proapoptotic BH3-only proteins NOXA and PUMA, subsequently priming AML cells to VEN-induced apoptosis [36]. Interestingly, the upregulated expression of the *PMAIP1* or *BBC3* genes, coding for NOXA and PUMA proteins, respectively, is not epigenetically regulated, as shown by the absence of methylation changes on DNA promoters and the rapid kinetics of apoptosis induction (<24 h). It appears to be an off-target of the drug, depending on the integrated stress response (ISR) induction and mediated by the ATF4 transcription factor [37]. The synergistic effect of the two treatments in vitro may also be related to the fact that the VEN–AZA combination targets the dependency of leukemic stem cells on the amino acid pathway [38].

## 3. Clinical Studies of VEN in AML

### 3.1. VEN Monotherapy

The first published clinical trial of VEN in patients with AML was a phase II single-agent study in predominately RR AML patients, in which 800 mg VEN was given daily. The median age was 72 years (range 19–84). This study showed a 19% overall response rate (ORR) and a median duration of remission of 48 days. Another 19% patients had a partial bone marrow response, with incomplete hematologic recovery. Rates of response were higher among patients with *IDH* mutations (*IDH*mut, 36%). VEN monotherapy showed an acceptable safety profile, with neutropenic fever being the most common (31%) grade 3/4 adverse event (AE) [39].

These encouraging signals of response led to the development of VEN combinations with other therapeutics, including low dose-intensity treatments, chemotherapy, and targeted agents. The main results of these clinical studies are summarized in Table 1.

### 3.2. Venetoclax in Combination with HMA

#### 3.2.1. Phase I Study

The non-randomized, open-label phase 1b M14-358 trial (NCT02203773) evaluated 400, 800, or 1200 mg VEN, daily in combination with either AZA (75 mg/m^2^, days 1–7 or DEC 20 mg/m^2^, days 1–5) in patients with ND AML ineligible for ICT. Patients who received prior HMA therapy and those with favorable-risk disease cytogenetics were excluded from the study. Median age was 74 years (range 65–86). Hematological and gastrointestinal (GI) AEs were the most common toxicities observed. Common serious (=grade 3/4) AEs included febrile neutropenia (43%), decreased WBC count (31%), anemia (25%), thrombocytopenia (24%), neutropenia (17%), and pneumonia (13%). Due to a higher frequency of hematological and GI AEs with the 1200 mg dose, only the 400 mg and 800 mg dose cohorts were expanded. The complete response (CR) rate was 30%, CR with incomplete recovery of blood counts (CRi) was 37%, ORR rate (CR + CRi + partial response (PR)) was 68%, and composite response rate (CR + CRi + PR + morphologic leukemia free state (MLFS)) was 83%. No difference in duration of response was seen between the 400 mg and 800 mg doses. With a median follow-up of 15.1 months, median OS for all groups was 17.5 months. Among the patients who received 400 mg VEN plus HMA, the CR + CRi rate was not different between AZA (71%) and DEC (74%) [40,41].

#### 3.2.2. Phase II Study

DEC has a short half-life, and pharmacodynamics studies suggested that 10-day exposure was more efficacious than 5-day administration. Ten-day DEC (DEC-10) showed a 40–64% ORR with a high response rate in patients with unfavorable risk cytogenetics or *TP53* mutations [56,57]. Based on these results, a single-center (MD Anderson) phase II trial was performed, postulating that DEC-10 may constitute a better backbone for VEN therapy than the 5-day DEC schedule used previously. Patients received 400 mg VEN from day-1 to day-28 and 20 mg/m^2^ DEC from day-1 to day-10 of induction. After reaching CR or CRi, patients received a 20 mg/m^2^ DEC maintenance for 5 days per cycle. VEN was stopped on day-21 if bone marrow showed aplasia or remission with less than 5% blasts. The use of FLT3 inhibitors (FLT3i) and IDH1/2 inhibitors (IDHi) was allowed in genotype-selected patients. The study included 168 patients with ND AML (*n* = 70), untreated secondary AML (sAML, *n* = 15), treated sAML (*n* = 28), and RR AML (*n* = 55). Median age was 71 years; ORR was 74% with 61% of patients obtaining CR or CRi. The most frequent serious grade AEs were neutropenic fever (38%), pneumonia (10%), and sepsis (10%) [42]. Ninety-seven patients (82%) achieving either a CR, CRi, or MLFS were included in the molecular residual disease (MRD) analysis. MRD-negative (MRDneg) status at 1, 2, and 4 months after starting therapy was achieved in 52% of patients and associated with a 37% cumulative incidence of relapse at 18 months and a 25 months median OS [43].

#### 3.2.3. Phase III Study (VIALE-A Trial)

The VIALE-A placebo-controlled randomized phase III trial included 431 patients (286 were allocated to the AZA + VEN 400 mg arm and 145 to the placebo + AZA arm). Twenty-five percent of patients had sAML. Patients with favorable cytogenetics were not excluded of the study; the median age was 76 years. CR was achieved in 36.7% and 17.9% of patients in the AZA + VEN arm and in the control arm, respectively (*p* < 0.001); CR + CRi was obtained in 64.7% and in 22.8% of patients (*p* < 0.001). Subgroup analyses showed a CR + CRi rate of 74.2% in the intermediate group and 52.9% in the poor-risk cytogenetics groups in the AZA + VEN arm versus 31.5% and 23.2% in the placebo arm. *IDH*mut were associated with better disease control with AZA + VEN than with the control. Median OS was 14.7 months versus 9.6 months (*p* < 0.001). Patients with an intermediate cytogenetic risk had a 20.8 months OS in the AZA + VEN group and 12.4 months in the control group, while those with a poor cytogenetic risk had a 7.6 months and 6.0 months OS, respectively [44]. Analyses of the 211 patients evaluable for the MRD showed that 78 patients (37%) achieved CR with MRDneg (<10^–3^, =0.1%). In this population, median survival was NR [58]. The most frequently reported serious AEs in the VEN + AZA arm and placebo + AZA arm were febrile neutropenia in 30% and 10%, pneumonia in 17% and 22%, sepsis in 6% and 8%, and hemorrhages in 9% and 6%, respectively.

Given its high efficacy and good toxicity profile, this treatment has become a new standard for unfit AML patients. Management of myelosuppression-related toxicities and dose adaptations, in particular for patients using drugs metabolized through cytochrome p450, have been detailed elsewhere [59,60,61].

### 3.3. Combination with LDAC

#### 3.3.1. Phase I/II Study

VEN was combined with LDAC in a pivotal phase Ib/II trial including >60-year-old patients with ND AML and ineligible for intensive chemotherapy. A prior HMA for myelodysplastic syndrome (MDS) antecedent was allowed; LDAC was given once daily at a standard dose of 20 mg/m^2^ for 10 days, and the VEN recommended phase II dose was 600 mg daily. Among the 82 included patients, 32% had poor-risk cytogenetics and 50% had sAML, 60% of which had prior HMA. The median age was 74 years (range 63–90). The most common hematological and non-hematological serious AEs were febrile neutropenia (42%) and GI toxicity (nausea, 70% and diarrhea, 49%), respectively. The CR + CRi rate was 54% (CR, 26% and CRi, 28%) and the MRDneg rate was 32%. The median OS was 10.1 months, with an estimated 1-year OS of 27%. Patients without prior HMA exposure had a longer median OS (13.5 months) than patients with AML previously exposed to HMA (4.1 months). Overall, these results do not appear to be inferior to the experience with VEN with HMA, especially when excluding patients with prior HMA [45].

#### 3.3.2. Phase III Study (VIALE-C)

Two hundred and eleven patients were included in the VIALE-C phase III randomized trial of LDAC, with or without VEN. Median age was 76 years (range 36–93) and 38% had sAML, of which half had prior HMA. A third of patients had poor-risk cytogenetics. The CR + CRi rates were 48% and 13% for the VEN + LDAC combination and LDAC alone, respectively (*p* < 0.001), with CR achieved in 27% and 7% of patients, respectively. The addition of VEN to LDAC resulted in a 25% survival benefit arm, which was not statistically significant (median OS was 7.2 months versus 4.1 months, *p* = 0.11). Nevertheless, an unplanned analysis with an additional 6 months of follow up, demonstrated statistical significance for OS, with a median of 8.4 months for the VEN arm versus 4.1 months (*p* = 0.04). The main serious AEs in the VEN arm were febrile neutropenia (32%), neutropenia (47%), and thrombocytopenia (45%). Induction mortality was 13% with VEN + LDAC and 16% with LDAC, possibly explained by the relatively high-risk population, as 60% were older than 75 years of age and 50% had ECOG performance status 2–3 [46].

Based on these two early-phase, open-label, non-randomized trials (NCT02203773-Venetoclax + HMAs, NCT02287233-Venetoclax + LDAC) results, the Food and Drug Administration (FDA) gave an accelerated approval for the use for the use of venclyxto® in combination with AZA for patients with untreated AML and ineligible for ICT. EMA-approval was based on the early-phase, open-label, non-randomized M14-358 trial (NCT02203773) and the VIALE-A trial.

### 3.4. HMA or LDAC-Based Combinations in Patients with RR AML

Few retrospective studies have evaluated the combination of VEN with HMA or LDAC in patients with RR AML. Global response rate was around 20% [62,63]. Prior therapy with a HMA also appears to be a risk factor for refractory disease [45,64].

### 3.5. Combination with Intensive Chemotherapy

Preclinical data based on in vitro BH3 profiling showed that blasts primed to apoptosis are highly sensitive to chemotherapy [65,66]. Moreover, interesting results of VEN-based treatment in patients with poor-risk cytogenetics or molecularly unfavorable groups led to the development of a combination between VEN and chemotherapy for high-risk patients, including elderly, sAML, or RR AML patients.

#### 3.5.1. High Dose Cytarabine + Idarubicin-Based Regimens

A phase I trial of escalating dose of VEN (200, 400, and 600 mg/day after 4 day ramp-up) in combination with standard 7 + 3 induction showed that 200 mg daily for 10 days can be safely given in adults patients with ND AML <60 years of age [47].

The Australian phase Ib study (CAVEAT study), using an attenuated 5 + 2 regimen combining CYTA and idarubicin (IDA), included patients ≥65 years (>60 years if monosomal karyotype) with ND AML. VEN was given in five dose-escalation cohorts of 50 to 600 mg daily for 14 days, including a 7-day dose ramp-up, followed by chemotherapy. Consolidation was given for up to 4 cycles consisting of 14-day VEN combined with 2-day CYTA and 1-day anthracycline chemotherapy. Maintenance with VEN was administrated in 14-day cycles every 28 days for 7 cycles. Fifty-one patients were included with a median age of 72 years (range, 63–80 years); 45% had sAML, and 31% had prior HMA exposure. The maximal tolerated dose (MTD) was not reached with VEN 600 mg in combination with 5 + 2 induction. Thirty-seven patients (72%) achieved ORR, with CR and CRi in 41% and 31%, respectively. The CR rate was 68% in de novo AML compared with 9% for sAML. The median OS for the entire cohort was 11.2 months. Poor OS was noted for patients with *TP53* mutations (3.6 months). The most common grade ≥3 AEs were febrile neutropenia (55%) and sepsis (35%). VEN doses needed to be reduced during consolidation phase to 400 mg/day because of hematological toxicity. The authors concluded that VEN can be safely administrated in combination with ICT [48].

#### 3.5.2. FLAG-IDA Regimen

The FLAG-IDA regimen combining Fludarabine (FLUDA), CYTA, IDA, and G-CSF support has been used in ND and RR AML in recent years. Composite CR rate (cCR = CR + CRi + CR with partial hematologic recovery (CRh)) was 85% in first line and 20–60% in the relapse settings [67,68]. The addition of VEN to FLAG-IDA was reported for patients with ND or RR AML and with high-risk MDS with >10% blasts. One or two inductions were administrated with VEN 400 mg 14-day. Owing to pronounced grade 3 and 4 neutropenia-related infectious complications and one DLT (typhlitis) in the original dose −1 level, the protocol was amended to evaluate an alternate dose −1 level, reducing the VEN induction duration to 14 days (from 21) and with attenuated CYTA. Four to six consolidation cycles were administrated with 7 days of VEN 400 mg and chemotherapy. Sixty-eight patients were enrolled (29 ND AML and 39 RR AML patients). Taken together, almost half of patients had adverse cytogenetics. Serious AEs included febrile neutropenia (50%), bacteremia (35%), pneumonia (28%), and sepsis (12%). ORR was 82% in the whole cohort (97% in the ND AML and 72% in the RR cohort). Composite CR was 76%; eighty-three percent of patients in cCR attained MRD negativity. The presence of *TP53* mutation was associated with inferior outcomes (cCR = 38%). EFS was 18 months in the whole cohort and 6.11 months in the RR cohorts of patients [49].

#### 3.5.3. CPX-351

CPX-351(Vyxeos®) is a dual-drug liposomal encapsulation of CYTA and daunorubicin (DAUNO) in an optimal 5:1 molar drug ratio. CPX-351 delivers drugs >24 h and persists in the bone marrow. CPX-351 was associated with a higher ORR (CR, 37.3%; CRi, 10.4%) than with the 7 + 3 control in the subgroup of sAML. Median OS was 9.5 months, versus 5.9 in the control arm [69]. A phase I/II study, enrolled patients in two cohorts of 18–65-year-old ND and >18-year-old RR AML patients. All patients were scheduled to receive an induction based on CPX-351 plus VEN 400 mg D2-D21 and up to four consolidation cycles with CPX-351 plus VEN D2-D21. Nevertheless, due to a high myelosuppression, VEN doses were decreased to 300 mg D2-D8. Data from 20 patients were reported at the ASH 2020 meeting. Median age was 51, and 45% of patients had adverse cytogenetics and 38% had *TP53* mutation. Response rate for the 18 evaluable patients was 44% (CR, 6%, CRi, 33%, MLFS, 6%). Median OS was 6.1 months [50].

Taken together, these two studies underline the hematological toxicity of VEN in combination with chemotherapy, which needs to be closely monitored and often necessitates dose adaptations.

#### 3.5.4. Cladribine-Based Regimen

The CLIA regimen consists in the incorporation of cladribine with idarubicin and high-dose CYTA as a frontline and salvage treatment for young patients (≤65 years) and was associated with a CR/CRi of 85%. The addition of a 7-day course of 400 mg VEN to the CLIA regimen was studied recently in a monocentric phase II study [51]. Eligible patients were 65 years or younger with a ND AML, mixed phenotype acute leukemia, or high-risk MDS with ≥10% blasts. Patients with a known *FLT3* mutation (*FLT3*mut) including internal tandem duplications (*FLT3*-ITD) or point mutations in the tyrosine kinase domain (*FLT3*-TKD) could receive MIDO or GILT during the induction and consolidation courses. The study included 50 patients; median age was 48 years. CR and ORR was 84% and 94%, respectively; 82% of the responding patients obtained MRDneg. At a median follow up of 13.5 months, the median OS and EFS were NR; estimated 12-month OS and EFS were 85% and 68%, respectively.

The MD Anderson phase II study combined VEN with a lower intensity regimen, alternating Cladribin, LDAC, and AZA. This regimen had previously been published without VEN and showed a CR rate of 58% and a CRi rate of 9% [70]. Inclusion criteria were ND AML >60 years patient or <60 years and unfit for chemotherapy. Forty-eight patients were treated in the study; 12 (25%) patients had sAML and 12 (25%) had adverse karyotypes. Median age was 68 years. Among the 48 evaluable patients presented in the abstract at the ASH 2020 meeting, 37 (77%) achieved a CR and 8 (17%) a CRi, for a CR/CRi rate of 92%. Thirty-six (80%) patients were negative for MRD with multi-parameter flow cytometry (FCM) at the time of CR/CRi. With a median follow-up of 11 months, the median OS was not reached, with 6- and 12-month OS rates of 86% and 70%, respectively. A grade 3 infection rate was reported for 25% of the cohort [52]. These results suggest that an intermediate-intensity regimen including VEN may be able to improve the disease control in comparison with standard AZA + VEN for high-risk patients unfit for ICT.

#### 3.5.5. Other Studies

Clinical trials combining VEN and chemotherapy including 7 + 3 (NCT03709758, NCT04062266), CPX-351 (NCT03826992, NCT04038437), and Gemtuzumab Ozogamycin (NCT04070768) are currently recruiting. Current studies recruiting patients with post induction AML are NCT04102020 (‘VIALE-M’) and NCT04062266. The treatment procedure consists in a combination of 14 to 28 days of VEN and 5 days of AZA. The VIALE-T study NCT04161885 is recruiting patients for post-HSCT consolidation with the same AZA + VEN regimen.

### 3.6. Combination with Targeted Agents

#### 3.6.1. IDH Inhibitors

Somatic mutations of *IDH1* or *IDH2* are present in 6–10% and 5–20% of patients, respectively [71]. *IDH* genes code for an enzyme with a crucial role in regulating tricarboxylic cycle fueling mitochondrial-dependent oxidative phosphorylation (OxPhos). Gain of function mutations of the genes confer a neomorphic enzymatic activity, by reducing α-KG to 2-hydroxyglutarate (2-HG), inducing oncogenic transcriptional deregulation [72]. Two IDHi have been recently approved for RR AML patients, namely ivosidenib (IVO) and enasidenib (ENA), for patients with *IDH1* and *IDH2* mutations, respectively. Both agents induced a durable response in 30–40% of patients and 10-month overall survival [73,74]. Preclinical data showed that *IDH*mut induces a BCL-2 dependency and a sensitization to VEN, explained by the (R)-2-HG-mediated inhibition of the activity of cytochrome c oxidase (COX) in the mitochondrial electron transport chain (ETC) [75]. These data are consistent with the initial findings of the highest sensitivity for *IDH*mut patients in clinical studies in monotherapy or in combination with HMA and LDAC [39,45,46,76]. In these cohorts, the median OS of *IDH*mutpatients was 19.4 months and 24.4 months, with LDAC and HMA, respectively (VIALE-A and VIALE-C).

A combination of AZA and IDH1i IVO without VEN has been recently reported in a phase Ib trial. In a total of 33 patients (median age 76 years), ORR was 78.3%; median OS was NR, with a median follow up of 16 months, and IDH1 clearance assessed by digital PCR was seen in 10/14 patients [77].

An interim analysis of the NCT03471260 phase I study, assessing the combination of VEN with IVO and AZA, was presented at the EHA 21 meeting. Eligible patients age ≥18 with *IDH1mut* MDS, ND AML or RR AML were enrolled into three dose levels (DL) cohorts: DL1 (IVO + VEN 400 mg), DL2 (IVO + VEN 800 mg), and DL3 (IVO + VEN 400 mg + AZA). Twenty-five evaluable patients were enrolled. Median age was 67 (range: 44–84). Eighty-four percent (*n* = 21) of patients had AML (ND, *n* = 13; RR, *n* = 8). The ORR was 92% (DL1: 67%, DL2:100%, DL3: 100%), with both non-responding patients (*n* = 2) treated within the DL1 cohort. Composite CR (CR + CRi + CRh) was 84%. One-year OS was 68% for the entire study population. MRD negativity by multiparameter FCM was obtained in 60%. Common serious AE included febrile neutropenia (28%) and pneumonia (24%). TLS and differentiation syndrome occurred in two and four patients, respectively [53].

Results of a phase II study were recently reported, showing the combination of AZA plus the IDH2i ENA in comparison with AZA without VEN (NCT02677922) in patients with *IDH2*mut ND AML [78]. A phase I study of a combination of VEN and ENA (NCT04092179) is currently recruiting, but no data have been presented so far. Nevertheless, in the phase II study studying the DEC-10 regimen plus VEN, the use of IDH inhibitors including ENA was allowed. No specific toxicities were reported [42].

#### 3.6.2. FLT3 Inhibitors

First generation FLT3i midostaurine (MIDO) and sorafenib (SORA) and second generation FLT3i gilteritinib (GILT) and quizartinib have shown interesting results in patients with FLT3mut that represent 30% of AML patients [71];. In these studies, the targeted therapies used as single agents were superior to standard treatments, in improving both response and OS [79,80]. Pre-clinical studies in *FLT3*mut cell lines, primary samples, and xenografts have shown a synergy between FLT3i and VEN through downregulation of MCL-1 and BCL-XL [81,82,83]. Prior clinical studies have demonstrated the safety and efficacy of the combination of SORA, quizartinib, or GILT with HMA, with an ORR of 65–80% and median OS 8.5–20 months [84,85,86,87].

A combination between VEN and GILT was reported in an updated interim analysis of a phase Ib clinical trial (NCT03625505), evaluating the combination in a cohort of RR AML. Thirty-nine *FLT3*mut patients were included in this cohort. Patients with prior GILT were excluded from expansion; prior VEN or other FLT3i was permitted. Patients received VEN 400 mg in combination with GILT 120 mg daily in 28-day cycles. Median age was 63 years (range 23–85). Most patients (*n* = 29; 74.4%) had received ≥2 prior lines of therapy, including ≥1 FLT3i. Composite CR + MLFS was achieved by 83.8% of patients, but this mainly included MLFS (54.1%). Febrile neutropenia was the most common serious AE (48.7%) [54].

A triplet therapy combining VEN, HMA, and FLT3i was recently published, combining FLT3i with the previously published regimen of DEC10-VEN [42]. The subgroup analysis of this trial included 25 *FLT3*mut patients (12 ND AML and 13 RR AML). FLT3i used in combination with DEC10-VEN were GILT (*n*  =  10), SORA (*n*  =  10), and MIDO (*n*  =  5). In ND patients, the CCR rate was 92%, with MRDneg by flow cytometry (FCM) in 56% and by PCR/NGS in 91% of responders. In RR AML the CCR rate was 62% with MRD negativity rate by FCM in 63% and by PCR/NGS in 100% of responders. Eight patients with RR AML had prior exposure to a FLT3i; the CCR rate was 63%, with *FLT3* PCR negativity in four out of four responding patients tested. The most frequent serious AEs was febrile neutropenia in 40% patients. After a median follow-up 14.5 months, the median OS was NR and 6.8 months for the ND and the RR patients, respectively [55].

Other trials testing similar triplet combinations of HMA, VEN with quizartinib (NCT03661307), and GILT (NCT04140487) in ND and RR AML are currently recruiting and will be useful for determining the optimal combinatorial approach.

## 4. Molecular Factors Driving Resistance

### 4.1. Genetic Factors

#### 4.1.1. TP53

Genetic studies have identified *TP53* mutation as a major factor influencing response to VEN [88,89,90]. An elegant study from the A. Wei lab in Melbourne, Australia, suggested that, despite good initial response after VEN treatment, the drug may select *TP53* mutated clones responsible for relapse by decreasing the expression of proapoptotic BH3-only proteins inducing BAX/BAK activation [91]. Consistently, clinical studies identified lower response and survival rates in patients with AML and *TP53* alterations [44,46,48]. A retrospective analysis of 121 patients treated with DEC10-VEN frontline therapy in a phase II trial was performed to specifically assess the role of *TP53* mutations [42,43,44,45,46,47,48,49,50,51,52,53,54,55,58,59,60,61,62,63,64,65,66,67,68,69,70,71,72,73,74,75,76,77,78,79,80,81,82,83,84,85,86,87,88,89,90,91,92]. Thirty-seven (31%) patients had *TP53*mut; patients with *TP53*mut were more likely to have therapy-related AML (43% versus 11%), adverse ELN cytogenetic risk in 92% compared to 27%. *TP53*mut patients had a lower rate of CR at 35% compared to 57% (*p* = 0.026), a lower rate of CR + CRi at 54% vs. 76% (*p* = 0.015), and lower rate of MRD negativity by FCM in 19%, compared to 52% in the *TP53*WT group (*p* = 0.001). Median OS was 5.2 months in patients with *TP53*mut, compared to 19.4 months in *TP53*WT AML.

#### 4.1.2. Other Genes

Mutation of *BAX*, one of the crucial proteins involved in MOMP, was found in 13.6% of a cohort of 44 VEN-resistant AML patients samples. In vitro recapitulation of the mutation was associated with a lower BAX protein expression and loss of apoptosis induction secondary to BH3-mimetics, but not conventional chemotherapy [93]. Contrary to CLL, in AML patients resistant to VEN, no mutations in BCL2 genes have been identified so far, possibly because CLL patients received a longer duration treatment. Indeed, the first detectable mutations occurred after 19 to 42 months of therapy, and their emergence anticipated clinical disease progression by many months [94].

### 4.2. Non-Genetic Factors

#### 4.2.1. BH3 Protein Expression and Occupation

Seminal in vitro studies found that VEN apoptotic cell death depends on BCL-2 family protein expression. High BCL2 expression was correlated with drug efficacy, which supported an on-target action of killing via competition for the BH3 binding site of BCL-2. On the contrary, the low expression of other key proteins involved in apoptotic regulation, such as BCL-XL or MCL-1, was negatively correlated with the drug efficacy [34]. Moreover, in early clinical studies, high BCL-2 /BCL-XL protein expression ratios was a prediction factor for longer response duration [32]. Priming of mitochondria for apoptosis sensibility depends on the functional collaboration between several antiapoptotic factors that can be, at least partially, independent of their level of expression. In a recent study using primary patient samples and patient-derived xenografts mice models, occupation of BH3 only proteins moved rapidly from BCL-2 to MCL-1, leading to VEN resistance. These studies, as well as others, support the use of MCL-1 inhibitors (MCL-1i) in combination with VEN, to reinforce the anti-leukemic effect [28,95,96].

#### 4.2.2. Cellular Differentiation

It has been observed that AML blasts with monocytic differentiation, as well as monocytic subclones, are more likely to be resistant to VEN monotherapy [97]. A recent retrospective study performed on 100 consecutive AML treated with AZA + VEN as a frontline therapy confirmed that monocytic AML patients (FAB-M5) were resistant in comparison with more undifferentiated AML. This correlates with a higher BCL-2/MCL-1 gene expression ratio in monocytic blasts, in comparison with undifferentiated blasts. Using single-cell RNA sequencing, this study also suggested that at relapse, patients samples were more enriched for blast with monocytic features and that these clones were already present at diagnosis and selected over the treatment [98].

#### 4.2.3. Metabolic Factors

Mitochondria are central hubs in the regulation of programmed cell death and cellular respiration, with one influencing the other. Studies from the C. Jordan Lab (University of Colorado, Denver, CO, USA) first discovered that the metabolic vulnerability of AML blasts could be leveraged by AZA + VEN. Focusing on a subpopulation of leukemic stem cells (LSC), they showed that LSC was highly dependent on OxPhos to survive, in comparison with the bulk compartment (and to their normal hematopoietic stem cells counterpart). This metabolic dependency was associated with a high level of BCL-2 expression and, consequently, sensitization to BCL-2 inhibition [99]. These in vitro findings have translational implications, as shown in a further study using mass spectrometry, metabolomics and single cell transcriptomics in patients samples [100]. Recent studies suggest that AML depends on various metabolic pathways to sustain a high OxPhos and proliferation capacity. For instance, AZA + VEN specifically target amino acid dependency in AML blasts, and it has been proposed that a switch toward fatty acid metabolism underlies VEN resistance [38,101,102]. Together, these studies point out the close interaction between cell metabolism and cell death, revealing the mitochondrial vulnerability of AML cells.

#### 4.2.4. Mitochondrial Structure and Machinery

CRISPR Cas9 screens on VEN resistant cell lines led to the uncovering of the close interaction between cellular metabolism and mitochondrial structure. This high throughput technology consists in performing multiple knock-out and/or knock-in on a cell population and applying selective pressure using drug treatments. Sequencing of the remaining cells after treatment identifies the genes involved in resistance and sensitivity toward the drug. A recent study using a CRISPR Cas9 screen identified CLBP, a key protein regulating cristae (the mitochondrial compartment in which OxPHOS takes place) as a key driver of VEN sensitivity. Consequently, genetic inhibition of this protein increased sensitivity to VEN in vitro and in vivo [90,103]. On the other hand, the genes found in genetically engineered resistant cell lines were mitochondrial ribosomal proteins, such as mitochondrial ribosomal protein L54 (MRPL54), mitochondrial ribosomal protein L17 (MRPL17), and ribosome binding factor A (RBFA), reflecting the mitochondrial ability to generate ATP to facilitate protein translation [104]. Finally, genes regulating heme biosynthesis are key factors regulating VEN sensitivity [105]. These studies have paved the way for innovative therapeutic combinations involving new drug strategies, such as those disturbing the mitochondrial metabolism or machinery.

## 5. Future Directions

**Figure 1:**
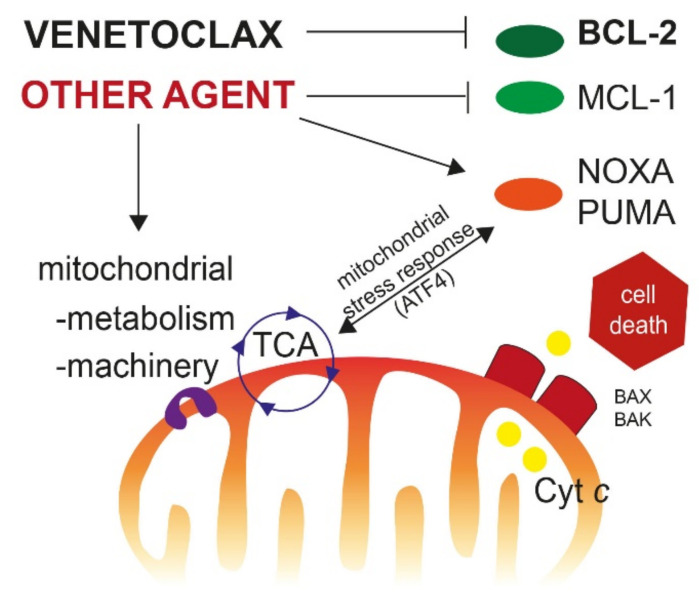
Main pathways for targeting venetoclax resistance. New therapeutic agents can inhibit other antiapoptotic proteins such as MCL-1 or increase the expression of proapoptotic BH-3 only proteins (NOXA, PUMA), in order to release cytochrome c, leading to apoptotic cell death. Targeting mitochondrial metabolism (i.e., OxPhos) or mitochondrial machinery (electronic transport chain, mitochondrial DNA replication, mitochondrial translation, mitochondrial structure, etc.) may increase the efficacy of BH-3 mimetics, by inducing the mitochondrial integrative stress response driven by the transcription factor ATF4. Abbreviation: TCA, tricarboxylic acid cycle. Cyt *c*: cytochrome *c*.

Induction of apoptotic cell death is the result of an imbalance between pro-survival (=antiapoptotic) signals and pro-death signals, mediated through proapoptotic proteins. The main strategies for treating resistance to BCL-2 inhibitors consist in inhibiting alternative antiapoptotic proteins, such as MCL-1, increasing proapoptotic signaling, and sensitizing cells to apoptosis; with all of these being interrelated (Figure 1).

### 5.1. Clinical Studies

A good example of the inhibition of an antiapoptotic agent is the co-treatment with a MCL-1i that re-sensitizes cells, by releasing the antiapoptotic brake. Preclinical data suggest that concomitant use of VEN + MCL-1i is strongly synergistic [28]. Current clinical trials involving MCL-1i AMG176 monotherapy (NCT02675452) or S64315 in combination with VEN (NCT03672695) are recruiting.

A second strategy consists in increasing the levels of proapoptotic proteins, such as NOXA or PUMA. This can be achieved with drugs such as AZA [36], pevonedistat, a novel agent that inhibits NEDD8-activating enzyme, or arsenic trioxide [36,106,107], which showed interesting preclinical activity in combination with VEN.

Finally, targeting mitochondrial metabolism to synergize with VEN is another promising approach, given the crucial role of mitochondria, both in cell death, and metabolism [101]. Links between metabolism deregulation and BH3-only protein expression are frequent. For instance, most of the drugs cited above share a common pathway of inducing a mitochondrial stress response mediated through the transcription factor ATF4, which is a direct activator of the BH3-only proteins NOXA and PUMA [37]. This is the case with CC90009, a first-in-class GSPT1-selective cereblon E3 ligase modulator. Degradation of GSPT1, an enzyme involved in protein translation, promoted the activation of the ATF4 pathway and subsequent apoptosis in AML cells [108]. A phase I clinical trial evaluating CC90009 in combination with AZA + VEN is currently recruiting (NCT04336982).

### 5.2. Preclinical Studies

It is likely that a new category of ‘mitochondrial’ drugs will be future candidates to overcome resistance in the clinical setting, in addition to or as a substitute for VEN.

Indeed, numerous preclinical studies have shown that targeting mitochondrial respiration or structure using small molecules can induce cancer cell death, including drugs targeting the ETC [109,110], mitochondrial translation [111,112], mitochondrial DNA replication [113], or mitochondrial Protease ClpP [114,115]. Using CRISPR Cas9 technology, we showed that ironomycin, a small molecule that sequesters iron into lysosome, was able to induce a dramatic reduction in mitochondrial iron, a disruption in OxPhos, and an ATF4-dependent mitochondrial stress, leading to a non-canonical apoptotic cell death. This lethality was highly synergistic with VEN in in vitro and in vivo AML models and in patient samples resistant to VEN (Garciaz S. et al., in review).

### 5.3. In Vitro Preclinical Screening

The development of experimental approaches will be key for the identification of the best VEN combination partners. This may be achieved using in vitro drug screening on primary patient samples collected at multiple time points (at diagnosis, at relapse, or during progression) after in vivo treatment by VEN, in order to test potential re-sensitization to BCL-2 or MCL-1 inhibitors. Simple assays measuring cytotoxicity are reliable options [116]. BH3 profiling may provide additional information, by detecting dependency on antiapoptotic proteins, as exemplified by the switch from BCL-2 toward MCL-1 dependency described recently [28]. Moreover, dynamic BH3 profiling can test the ability of a drug to induce apoptosis by modulating the level of mitochondrial priming. For instance, one could expect that new (or old, repositioned) drugs would modify the threshold triggering MOMP, through the upregulation of proaptototic proteins or by inducing metabolic or structural mitochondrial alterations, with one influencing another. It is likely that future personalized treatments in AML will involve a BH3-mimetics backbone treatment, upon which new partners will be added, depending on the intrinsic apoptosis ability of patient cells. Finally, a new area of research will be to target the non-apoptotic pathways of cell death, which will induce a synthetic lethality in patients resistant to canonical apoptosis

## 6. Conclusions

Non-intensive approaches are rarely curative in AML. Historically, HMA and LDAC were associated with a 20–30% response rate and a 10-month median OS. The development of VEN-HMA based combination regimens has revolutionized the treatment of these aggressive diseases, as it has led to a doubling of response rates and a significant increase in survival. Despite these good results, relapses and primary refractory diseases are still a matter of concern. Triplet therapies based on the combination of VEN + HMA (or LDAC) + targeted agents may represent good options for patients with targeted molecular alterations. For the vast majority of patients, such alterations are lacking, and new drugs restoring proapoptotic imbalance are under development. Besides the tremendous progress achieved with VEN in low-intensity therapies, VEN may also be incorporated into intensive regimens. Preliminary results have shown the feasibility of such combinations, and their efficacy is currently being assessed. Finally, the experience with VEN opens the way for the development of treatment approaches targeting, not only the BCL-2 family members, but also the close connection between metabolism and the cell death pathways.

## Figures and Tables

**Table 1:** Main studies evaluating venetoclax efficacy in AML, as monotherapy or in combination.

Combination Therapy	Phase; Name; ID	Study Population	Venetoclax Doses	Other Agents Administration Regimens	Response Rate	Survival Rate	Ref
no	I; NCT01994837	RR AML	800 mg D1 to D28	no	19% (ORR)	4.7 months (median OS) 2.3 months (median LFS)	[39]
HMA	I; (M14-358); NCT02203773	ND AML ineligible for chemotherapy	400–800–1200 mg D1 to D28	AZA 75 mg/m^2^, D1 to D7 or DEC 20 mg/m^2^, D1 to D5	37% (CR), 68% (ORR) 83% (cCR)	17.5 months (median OS)	[40,41]
	II; NCT03404193	ND patients with AML > 60 yrs RR patients > 18 years	400 mg D1 to D28 (D1 to D21 if blasts < 5%)	DEC 20 mg/m^2^, D1 to D10 (induction) DEC 20 mg/m^2^, D1 to D5 (consolidation)	63% (CR + CRi), 74% (ORR)	18.1 months (median OS)	[42,43]
	III; (VIALE-A); NCT02993523	ND AML ineligible for chemotherapy	400 mg D1 to D28 vs. placebo	AZA 75 mg/m^2^, D1 to D7	36.7% vs. 17.9% (CR) 64.7% vs. 22.8% (CR + CRi)	14.7 months vs. 9.6 months (median OS)	[44]
LDAC	I/II; NCT02287233	ND AML ineligible for chemotherapy	600 mg D1 to D28	LDAC 20 mg/m^2^ days D1 to D10	26% (CR) 28% (CRi)	10.1 months (median OS)	[45]
	III; (VIALE-C); NCT03069352	ND AML ineligible for chemotherapy	600 mg D1 to D28 vs. placebo	LDAC 20 mg/m^2^ D1 to D10	27% vs. 7% (CR) 21% vs. 6% (CRi)	7.2 months vs. 4.1 months (median OS)	[46]
Anthracyclin-based chemotherapy	I	ND AML <60 years	200 to 600 mg D1 to D11	DAUNO 60 mg/m^2^/d D2 to D4 CYTA 200 mg/m^2^/d D2 to D8	ND	ND	[47]
	Ib; (CAVEAT); ACTRN12616000445471	ND AML ≥ 65 years (>60 years if monosomal karyotype)	Induction 50 to 600 mg D-6 to D7	CYTA 100 mg/m^2^/d D1 to D5 IDA 12 mg/m^2^/d D2 to D3	72% (ORR) 41% (CR) 31% (CRi)	11.2 months (median OS)	[48]
			Consolidation 50 to 600 mg (4 cycles) D-6 to D7	CYTA (bolus) 100 mg/m^2^/d D1 to D2 IDA 12 mg/m^2^/d D1			
			Maintenance (7 cycles) 50 to 600 mg D1 to D14	no			
	Ib/II; NCT03214562	ND, RR AML or high-risk MDS with > 10% blasts	Induction (1 or 2) 400 mg D1 to D14	modified FLAG-IDA FLUDA 30 mg/m^2^ D1 to D14 CYTA 1.5 g/m^2^ D2 to D6 IDA 8 mg/m^2^ D4 to D6 (ND AML) or 6 mg/m^2^ D5 to D6 (RR AML)	82 % (ORR) 37% (CR) 15% (CRh) 7% (CRi)	NR (median OS), 18 months (median EFS)	[49]
	II; NCT03629171	18–65 years ND >18 years RR AML	400 mg D2 to D22 (decreased to 300 mg D2 to D8 due to toxicities)	induction, CPX-351 D1, D3, D5	44% (ORR) 6% (CR) 33% (CRi) 6% (MLFS)	6.1 months (median OS)	[50]
Cladribin-based chemotherapy	II; NCT02115295	ND AML < 65 years	400 mg D2 to D8	Induction: Cladribine 5 mg/m^2^ D1 to D5 CYTA (1.5 g/m^2^ for pts < 60 years and 1 g/m^2^ for pts aged ≥ 60 years) D1 to D5 IDA 10 mg/m^2^/d D1 to D3	84% (CR) 94% (ORR)	NR (median OS and EFS)	[51]
				Consolidation, Cladribine 5 mg/m^2^ D1 to D3 CYTA 1 g/m^2^ (for patients aged <60 yrs) and 0.75 g/m^2^ (for patients aged ≥60 yrs) D1 to D5, IDA 8 mg/m^2^/d D1 to D2			
	II; NCT03586609	ND AML > 60 years or <60 years unfit for conventional chemotherapy	Induction 100–400 mg D1 to D21	Cladribine 5 mg/m^2^ D1 to D5 LDAC 20 mg/m^2^ D1 to D10	78% (CR) 15% (CRi)	NR (median OS)	[52]
			consolidation VEN 100–400 mg D1 to D14 (Patients with MRD negativity received only 7 days of VEN)	Alternance of 2 cycles of A and B A, Cladribine 5 mg/m^2^ D1 to D3; LDAC 20 mg/m^2^ D1 to D10 B, AZA 75 mg/m^2^ D1 to D7			
			consolidation VEN 100–400 mg D1 to D14 (Patients with MRD negativity received only 7 days of VEN)	Alternance of 2 cycles of A and B A, Cladribine 5 mg/m^2^ D1 to D3; LDAC 20 mg/m^2^ D1 to D10 B, AZA 75 mg/m^2^ D1 to D7			
IDH inhibitor (ivosidenib)	Ib/II; NCT03471260	>18 years old *IDH1*mut MDS, ND secondary AML, or RR AML	Cohort 1 IVO + VEN 400 mg D1 to D14 Cohort 2 IVO + VEN 800 mg Cohort 3 IVO + VEN 400 mg + AZA	IVO; 500 mg daily from D15, +/− AZA; 75mg/m^2^ D1 to D7 every 28 days.	92% (ORR), 84% cCR (CR + CRi + CRh)	NR (median OS). 68% (1-year OS)	[53]
FLT3 inhibitors	Ib; NCT03625505	RR *FLT3*mut AML	400 mg D1 to D28	GILT 150 mg D1-D28	83.8% (modified cCR = CR + CRi + CRh + MLFS),	5.1 months (median EFS)	[54]
	II; NCT03404193	ND patients with AML >60 years RR patients >18 years	DEC10-VEN	GILT, SORA and MIDO at recommended doses	92% (cCR, ND AML), 62% (cCR, RR AML)	NR (median OS in ND AML) 6.8 months (median OS in RR AML)	[42,55]
